# Remifentanil for modified rapid sequence induction in children: a proof-of-concept study in a selected pediatric population

**DOI:** 10.1016/j.bjane.2026.844837

**Published:** 2026-09-24

**Authors:** Julien Pico, Chrystelle Sola, Laurent Hertz, Julien Riera, Christopher Scott, Kévin Chapron, Philippe Pirat, Sophie Bringuier, Christophe Dadure

**Affiliations:** a Montpellier University Hospital, Department of Maternal, Child and Women's Anaesthesiology and Intensive Care Medicine, Paediatric Anaesthesia Unit, Montpellier, France; b University of Montpellier, Debrest Institute of Epidemiology and Public Health IDESP, UMR INSERM, Montpellier, France; c University of Montpellier, CNRS, INSERM, Institute of Functional Genomics, Montpellier, France; d Montpellier University Hospital, Department of Medical Statistics and Epidemiology, Montpellier, France

**Keywords:** Airway management, Atropine, Intubation, intratracheal, Pediatric anesthesia, Rapid sequence induction and intubation, Remifentanil

## Abstract

**Introduction:**

Rapid Sequence Induction (RSI) is widely used in pediatric anesthesia for children at risk of pulmonary aspiration. Modified RSI has been proposed to reduce the risk of hypoxemia during induction while maintaining airway protection. This proof-of-concept study aimed to describe peri-intubation outcomes associated with the use of intravenous bolus remifentanil during modified RSI in a selected pediatric population at risk of pulmonary aspiration.

**Methods:**

This proof-of-concept study analyzed data from pediatric patients, with at least one risk factor for pulmonary aspiration, undergoing modified RSI with remifentanil between December 2021 and August 2023. Exclusion criteria included the planned use of a neuromuscular blocking agent for RSI, preoperative hypoxemia, hemodynamic shock or difficult intubation risk factors. Remifentanil was administered by direct intravenous injection with optional prophylactic administration of atropine. Data on intubation success rates, major and minor complications and rescue treatment were collected and analyzed.

**Results:**

A total of 267 children were included (median age 8.0 years [IQR 5.0–12.0]). Successful first-attempt intubation without major complications was achieved in 92.9% (248/267; 95% CI 88.9–95.6). No major complications were reported. Nineteen patients (7.1%) required more than one intubation attempt. In multivariable analysis, age < 1-year (adjusted OR = 11.4, 95% CI 3.5–34.3; p < 0.001) and Cormack–Lehane grade ≥ 2 (adjusted OR = 10.2, 95% CI 1.8–57.2; p = 0.008) were associated with failed first-attempt intubation. Minor complications occurred in 15.7% of patients, mainly transient hypotension.

**Conclusion:**

In this proof-of-concept study of a selected pediatric population, modified RSI with remifentanil was associated with a first-attempt intubation success rate of 92.9%, with no major complications observed. These descriptive findings should be considered hypothesis-generating and support further evaluation of remifentanil-based modified RSI in prospective randomized studies.

## Introduction

As pulmonary aspiration remains a potentially devastating complication of General Anesthesia (GA) in all age groups,^1^ Rapid Sequence Induction (RSI) is a standard procedure for tracheal intubation in children at risk of aspiration or regurgitation of gastric contents. Current data from the APRICOT study^2^ show that almost 11% of pediatric general anesthesia is performed by RSI, making this a regular practice of pediatric anesthesiologists.

As in adults, the aim of RSI in the pediatric population is to achieve adequate intubation conditions and to minimize the delay between loss of consciousness and airway protection by tracheal intubation. Reduced apnea tolerance combined with reduced functional residual capacity^3^ in children leads to an increased risk of hypoxemia: the younger the child, the greater the risk of desaturation.

The French guidelines, entitled “*Management of the child’s airway under anesthesia*”,^4^ recommend using a rapid intravenous neuromuscular blocking agent during a conventional RSI (Grade 1+), with succinylcholine as the first choice. Experts introduce the concept of a “modified” or “controlled” RSI with a gentle mask ventilation not exceeding 15 cm H_2_O to maximize oxygen reserves and to reduce the risk of hypoxemia before intubation.

Several authors suggested that modified RSI may better balance the risks of pulmonary aspiration and hypoxemia in pediatric anesthesia.^5^^,^^6^ Modified RSI therefore represents an adaptation of conventional RSI intended to reduce oxygen desaturation during induction while maintaining airway protection strategies. The choice of neuromuscular blockade during RSI remains a separate consideration, and current guidelines continue to recommend the use of a rapid-acting neuromuscular blocking agent.^4^^,^^7^^,^^8^

Although rapid-acting neuromuscular blocking agents remain recommended during RSI, remifentanil has been used in our institution as part of a modified RSI strategy in selected pediatric patients without routine neuromuscular blockade.

Remifentanil is an ultra-short-acting opioid with rapid elimination through plasma esterases and favorable pharmacokinetic properties in children, particularly in neonates.^9^

Remifentanil used as an IV injection of 3 to 4 µg.kg^-1^ appears to offer optimal intubation conditions based on a scoring system^10^ in a pediatric population without causing severe cardiovascular depression or signs of muscular rigidity.^11^ However, previous pediatric studies excluded emergency procedures and children at risk of pulmonary aspiration.

The aim of this study was to describe peri-intubation outcomes associated with intravenous bolus remifentanil during modified RSI in a selected pediatric population, assessed by first-attempt intubation success and the occurrence of peri-intubation complications.

## Methods

### Study design

We conducted a retrospective analysis of prospectively recorded data, in the department of pediatric anesthesia. Anonymized anesthesia data from children who received remifentanil for modified RSI between December 2021 and August 2023 were collected.

### Population

Children aged up to 18 years old requiring RSI were eligible for enrolment. For the present retrospective analysis, we included consecutive children who received IV remifentanil bolus for a modified RSI procedure, with at least one of the following risk factors for pulmonary aspiration: bowel obstruction, recent vomiting or nausea, post traumatic ileus, severe chronic gastric dysfunction, insufficient preoperative fasting period (less than 6 hours). Exclusion criteria included the planned use of neuromuscular blocking agent for RSI, preoperative hypoxemia, hemodynamic shock, or difficult intubation risk factors.

### Usual per operative procedures

On arrival to the operating room, an IV catheter was inserted, if not already present. Baseline blood pressure, heart rate and pulse oximetry were routinely recorded. Preoxygenation was performed with 100% oxygen using a tightly fitting face mask until an adequate end-expiratory oxygen concentration was achieved, according to institutional practice and current pediatric airway management guidelines. Remifentanil was administered as a rapid intravenous injection following hypnotic administration. No predefined duration or specific administration rate was standardized in this observational setting. Laryngoscopy was generally performed approximately 60 seconds after induction, once loss of consciousness and adequate intubating conditions had been achieved. Gentle mask ventilation is part of the modified RSI approach and may be performed at the discretion of the anesthesiologist during induction. When applied, inspiratory pressure was limited to 15 cm H_2_O. The use and frequency of mask oxygenation were not specifically recorded in the database. Intubation was performed by senior anesthesiologists, anesthesia residents with more than one year of training, or nurse anesthetists. Residents performed laryngoscopy under the direct supervision of a senior pediatric anesthesiologist. The choice to use prophylactic IV atropine (20 µg.kg^-1^) during induction to prevent bradycardia was made by the anesthesiologist in charge.

In the event of an early complication (within ten minutes of RSI), the use of rescue drugs or emergency ventilation techniques was observed.

### Collected data

All data included in the study come from the “Vigilance Anesthetic in the PeriOperative period” (VAPO) questionnaire. The VAPO is a prospective collection of perioperative anesthetic data, integrated directly into the computerized patient chart. The VAPO has been used in our pediatric center for several years for all anesthesia procedures to document the perioperative anesthetic management, monitor patients at risk of complications and track post-operative outcomes. A special section was added to obtain data on the use of IV bolus remifentanil in the RSI procedure.

The study was based on prospectively collected data routinely recorded in the institutional VAPO database, an institutional quality assurance registry used for all pediatric anesthesia procedures as part of standard clinical practice and quality monitoring. Approval for the retrospective scientific analysis of these anonymized prospectively collected data was obtained in April 2022 (IRB Montpellier University Hospital – IRB-MTP_2022_04_202201075). No consent collection was required for this retrospective observational study.

Patient characteristics included: age, sex, weight, American Society of Anesthesiologists classification (ASA). Surgery data included the timing of surgery (urgent or scheduled) and the type of surgery (urologic, digestive, orthopedic, neurosurgery or other). Intubation data included: risk factors for pulmonary aspiration, induction drugs and their doses, use of prophylactic atropine during induction, use of emergency drugs after intubation. The use of direct or indirect laryngoscopy, Cormack-Lehane grade, number of laryngoscopies attempts prior to intubation, vocal cord position during the intubation procedure, cough response during intubation and operator satisfaction (very satisfied, satisfied, moderately satisfied or unsatisfied) were also reported.

### Outcomes

The primary outcome was successful tracheal intubation at the first attempt without major complications following modified RSI with IV bolus remifentanil used in combination with a hypnotic agent. A successful intubation at the first attempt was defined as a successful endotracheal tube placement during the first laryngoscope insertion. Complications were collected up to ten minutes after the administration of induction drugs. Major complications were defined as: hypotension requiring more than one injection of ephedrine and IV crystalloid administration (20 mL.kg^-1^), bradycardia requiring more than one dose of IV atropine (20 µg.kg^-1^), hypoxemia below 90% requiring face mask ventilation prior to intubation or manual ventilation following intubation, pulmonary aspiration. Minor complications were defined as: an episode of hypotension reversible after just one injection of ephedrine or IV crystalloid fluid administration (10 mL.kg^-1^), an episode of reversible bradycardia after a single dose of atropine or oxygen desaturation below 90%, quickly reversible after intubation and initiation of ventilation.

The secondary outcomes were to identify factors associated with failed first-attempt intubation and to describe peri-intubation complications.

### Statistical analysis

#### Primary outcome

The proportion of successful tracheal intubations at the first attempt without major complications was presented as percentage and its 95% Confidence Interval (95% CI).

#### Secondary outcomes

Failed intubation at first attempt: To evaluate factors associated with failed intubation at first attempt, a univariate analysis was performed on patient characteristics, the timing and the type of the surgery, remifentanil and propofol doses and use of prophylactic atropine. Count data, such as the number of laryngoscopy attempts, were compared using Poisson regression. The comparison between groups (success *vs* failed at first attempt) on categorical data were performed using the χ^2^ test or the Fisher exact test, as appropriate. Continuous data were compared with the Wilcoxon test. Because of the limited number of first-attempt failures, multivariable logistic regression was restricted to a parsimonious model including three clinically selected covariates: age (< 1-year vs. ≥ 1-year), Cormack-Lehane (grade ≥ 2 vs. grade 1) and remifentanil (dose < 3.1 vs. ≥ 3.1). Associations with failed intubation at first attempt were presented as adjusted Odds Ratios (OR) with 95% confidence intervals.

Descriptive continuous variables were presented as means ± SD or medians (1Q, 3Q) and categorical data as numbers (%). p-values below 0.05 were considered statistically significant. The statistical analysis was carried out using SAS Enterprise version 8.3 (SAS Institute, Cary, NC).

## Results

We included 267 children at risk for pulmonary aspiration who underwent general anesthesia using a modified RSI procedure.

The median age was 8.0 years (IQR 5.0–12.0). Age ranged from one day to sixteen years, including 21 (7.9%) children younger than 1-year. Patient characteristics are presented in Table 1. The main risk factors for pulmonary aspiration were bowel obstruction, recent vomiting or post traumatic ileus, reported in 81.3% of cases (217 patients). Digestive or urologic surgery was the most frequent procedure, reported in 56.9% of cases (152 patients). The hypnotic agent used in combination with remifentanil was propofol in all patients. The median (IQR) dose of bolus remifentanil was 3.1 (2.9‒3.5) µg.kg^-1^. Direct laryngoscopy was used in 208 cases (78.2%).

**Table 1:** Patient characteristics.

Characteristics	Remifentanil n = 267
Age, median (Q1, Q3), years	8.0 (5.0–12.0)
< One year n (%)	21 (7.9)
≥ One year n (%)	246 (92.1)
Male n (%)	172 (64.4)
Weight, median (Q1, Q3), kg	26.5 (18.0–41.3)
American Society of Anesthesiologists Physical Status Classification System score (ASA) n (%)	
I	179 (67)
II	53 (19.9)
III	33 (12.4)
IV	2 (0.8)
Timing of surgery n (%)	
Urgent or semi-urgent	249 (93.3)
Scheduled	18 (6.7)
Type of surgery n (%)	
Digestive, urologic	152 (56.9)
Orthopedic	73 (27.3)
Others: Neurosurgery, endoscopic procedures	42 (15.7)
Risk factors for pulmonary aspiration n (%)	
Bowel obstruction, recent vomiting	154 (57.7)
Post-traumatic ileus	63 (23.6)
Others: severe chronic gastric dysfunction, insufficient preoperative fasting period	50 (18.3)
Remifentanil dose, median (Q1, Q3), µg.kg^-1^	3.1 (2.9–3.5)
Propofol dose, median (Q1, Q3), mg.kg^-1^	6.0 (5.1–7.5)
Prophylactic atropine n (%)	114 (42.7)
Laryngoscopy n (%) (n = 266)	
Direct Laryngoscopy	208 (78.2)
Video Laryngoscopy	58 (21.8)
Cormack-Lehane grade n (%) (n = 266)	
1: Full glottis view	258 (97)
2: Partial view of the glottis	7 (2.6)
3: Only epiglottis seen, no view of the glottis seen	1 (0.4)

Values are presented as mean ± SD, median (Q1, Q3), or number (%).

Successful intubation at the first attempt without major complications was accomplished in 92.9% (95% CI 88.9‒95.6) of children (248/267 patients). No major complications were observed during the study period.

Patient and procedural characteristics according to first-attempt tracheal intubation success are presented in Table 2. Intubation failed at the first attempt in 19 children (7.1%). Successful intubation required two and three attempts in 16 (6.0%) and 3 (1.1%) patients, respectively. In the multivariable analysis, infant age < 1-year (adjusted OR = 11.4; 95% CI 3.5–34.3; p < 0.001) and Cormack–Lehane grade ≥ 2 (adjusted OR = 10.2; 95% CI 1.8–57.2; p = 0.008) were associated with failed first-attempt intubation, whereas remifentanil dose ≥ 3.1 µg.kg^-1^ was not (adjusted OR = 1.3; 95% CI 0.45–3.7; p = 0.63).

**Table 2:** Patient and procedural characteristics according to first-attempt tracheal intubation success.

Characteristics	Successful intubation at the first attempt (n = 248)	Failure intubation at the first attempt (n = 19)	p-value
Age, median (1Q, 3Q), years	8.00 (5.00; 12.00)	1.00 (0.08; 8.00)	< 0.01
< One year n (%)	13 (5.24)	8 (42.11)	< 0.01
Weight, median (1Q, 3Q), kg	27.10 (18.80; 41.70)	10.61 (3.83; 20.75)	< 0.01
Male n (%)	157 (63.31)	15 (78.95)	0.17
ASA score n (%)			< 0.01
I	172 (69.35)	7 (36.84)	
II	48 (19.35)	5 (26.32)	
III	27 (10.89)	6 (31.58)	
IV	1 (0.40)	1 (5.26)	
Timing of surgery n (%)			0.13
Scheduled	15 (6.05)	3 (15.79)	
Urgent or semi-urgent	233 (93.95)	16 (84.21)	
Type of surgery n (%)			0.04
*Orthopedic surgery*	72 (29.03)	1 (5.26)	
*Digestive, urologic surgery*	139 (56.05)	13 (68.42)	
*Other surgeries*	37 (14.92)	5 (26.32)	
Risk factors for pulmonary aspiration n (%)			
*Bowel obstruction, recent vomiting*	140 (56.45)	14 (73.68)	0.14
*Post-traumatic ileus*	62 (25.00)	1 (5.26)	0.05
*Other risk factors*	46 (18.55)	4 (21.05)	0.76
Direct laryngoscopy n (%)	194 (78.54)	14 (73.68)	0.57
Cormack-Lehane grade n (%)			< 0.01
1	243 (98.38)	15 (78.95)	
2	4 (1.62)	3 (15.79)	
3	0 (0.00)	1 (5.26)	
Prophylactic Atropine n (%)	104 (41.94)	10 (52.63)	0.36
Remifentanil dose, median (1Q, 3Q), µg.kg^-1^	3.10 (2.90; 3.40)	3.40 (2.80; 3.90)	0.36
< 3.1 µg.kg^-1^ n (%)	136 (54.84)	12 (63.16)	0.48
Propofol dose, median (1Q, 3Q), mg.kg^-1^	6.00 (5.10; 7.50)	6.00 (4.20; 7.00)	0.33

Values are presented as median (Q1, Q3), or number (%).

Vocal cords were in the abducted position in 245/264 (92.8%) children, and airway reactivity during tube passage was noted in 67/260 (25.8%) children. Operators rated intubation conditions as very satisfactory in 210/257 (81.7%) children and satisfactory in 39/257 (15.2%) children.

Forty-two (15.7%) children had at least one minor complication during the first 10 minutes following induction. Thirty-eight (14.2%) patients presented an episode of minor hypotension, which was corrected by a single injection of ephedrine without IV crystalloid fluid administration. Minor hypotension occurred in 1 of 21 children aged less than one year (4.8%) and in 37 of 246 children aged one year or older (15.0%). Three episodes (1.1%) of bradycardia were recorded in older children: two were 13 years old and the third was 16 years old. Two episodes of bradycardia were associated with minor hypotension. No episode of bradycardia was observed in children aged less than one year. Three patients (1.1%) presented an episode of desaturation below 90%. All were aged less than one year. For two children, the VAPO reported an episode of bronchospasm linked to selective intubation, quickly corrected by adjusting the tracheal tube position. The third episode of desaturation was linked to difficulties with tracheal intubation, requiring a change of operator.

## Discussion

To our knowledge, this study represents one of the first observational cohorts evaluating remifentanil in combination with propofol for modified RSI in pediatric patients at risk of pulmonary aspiration. The results of this study showed a high success rate of 92.9% for tracheal intubation at the first attempt without major complications. These findings provide preliminary descriptive data regarding peri-intubation outcomes associated with this modified RSI approach in a selected pediatric population.

Few studies have evaluated the use of IV bolus remifentanil for modified RSI in pediatric patients, particularly in children at risk of pulmonary aspiration. Previous studies,^10^^,^^11^ which reported excellent intubation conditions with remifentanil used in pediatrics, systematically excluded emergency procedures, often associated with a potential risk of aspiration. In addition, isolated pediatric case reports have previously described the use of propofol-remifentanil combinations for RSI in children at risk of aspiration.^12^ Blair et al.^10^ showed that remifentanil at a dose of 3 µg·kg^-1^ provides similar intubation conditions to mivacurium. In the same way, Klemola et al.^11^ showed that excellent intubation conditions were more frequent in the group receiving IV remifentanil at a dose of 4 µg.kg^-1^ than in the rocuronium group. In accordance, the average dose of bolus remifentanil used in the present study was 3.19 ± 0.66 µg.kg^-1^. In addition, in the current study, the selected population was children at risk of aspiration, for whom practitioners were free to choose a classical or modified RSI protocol, including, or not, the planned use of a neuromuscular blocking agent. In the modified RSI current cohort, this study reported a successful tracheal intubation at the first attempt in 92.9% of patients. Previous pediatric emergency RSI cohorts have reported variable first-attempt success rates, including 78% in a multicenter registry and 52% in a single-center study using systematic video review.^13^^,^^14^ However, no direct comparison can be made given the important differences in study design, patient populations, clinical settings, and airway management strategies across studies.

Regarding intubation conditions, while the vocal cords were in the abducted position in 92.8% of children, airway reactivity during tube passage through the vocal cords was observed in 25.8% of cases. The VAPO questionnaire did not distinguish between mild airway reactivity, coughing during tracheal tube passage, or reactivity related to cuff inflation. These results appear to be consistent with the study by Hanna et al.^15^ who reported no difference in the occurrence of a cough reaction between intubations with suxamethonium 1.2 mg.kg^-1^ and remifentanil 4 µg.kg^-1^. Acceptable intubation conditions have been shown to increase with increasing doses of remifentanil.^10^^,^^16^ Considering the pharmacological properties of remifentanil, both the administered dose and timing between drug administration and laryngoscopy may influence airway reactivity. The time interval between remifentanil administration and laryngoscopy was not recorded in this study, which limits interpretation. Variability in this interval may have contributed to the observed airway reactivity. These findings should be taken into account when interpreting intubating conditions in this cohort and suggest that airway reflex suppression may be inconsistent under the conditions observed in this study.

In a recent adult study by Grillot et al.^17^ remifentanil was compared to suxamethonium for RSI. The remifentanil group did not meet the criterion for non-inferiority regarding successful intubation without major complications (66% vs. 71% in the remifentanil group vs the neuromuscular blocking agent group, respectively). RSI failure was attributed to the inability to intubate on the first attempt, while episodes of major hemodynamic instability, prolonged arrhythmia > 30s or cardiac arrest were reported in 20% of patients with similar rates in the remifentanil and neuromuscular blocking agent groups. Although a high first-attempt intubation success rate was observed in the present cohort, the uncontrolled design of this study does not permit any comparison with alternative RSI strategies. As previously indicated, the pediatric population is significantly distinct from the adult population. Children differ from adults in terms of airway anatomy, oxygen reserve, apnea tolerance, and hemodynamic responses during induction of anesthesia, which may influence both intubation conditions and complication profiles during modified RSI. In the present study, episodes of mild and transient hypotension were the most frequent minor complications, observed in 14.2% of cases. All episodes were corrected by a single injection of ephedrine without IV crystalloid fluid administration. Previous pediatric studies by Blair et al.^10^ and Klemola et al.^11^ systematically administered prophylactic IV atropine (at doses of 10 µg.kg^-1^ or 15 µg.kg^-1^) when using remifentanil for induction, did not report hypotension or bradycardia requiring treatment. It should be noted, however, that there is currently no consensus on what constitutes hypotension in the literature on pediatric anesthesia. Wani et al.^18^ studied variations in blood pressure in children aged 2 to 8 years in the 12 minutes following the induction of anesthesia to investigate the incidence of post-induction hypotension. The incidence varied considerably based on the definition used, 27.5% if the 5^th^ percentile of the systolic blood pressure for age was used versus 57.2% (107 of 189 patients) when a 20% decrease of the systolic blood pressure from baseline was used. In adult classical RSI, a high rate of cardiorespiratory complications was reported, with 18% experiencing hypotension after GA induction.

Finally, infant age < 1-year and Cormack-Lehane grade ≥ 2 were associated with failed first-attempt intubation in the multivariable analysis. These findings are consistent with the pediatric airway literature, which indicates that infants and children with more difficult laryngoscopic views are at increased risk of intubation failure.^19^

Therefore, these results provide additional descriptive data regarding the use of remifentanil for modified RSI in selected pediatric patients managed by teams experienced with this technique.

Limitations of the present study include the absence of a control group, the retrospective analysis of prospectively collected data, and a potential selection bias. Patients with known difficult airway risk factors were excluded, which may explain the high proportion of favorable Cormack-Lehane grades observed in this cohort. This selection process may also have contributed to the high first-attempt intubation success rate observed in the present study and may limit the generalizability of the findings to broader pediatric populations. Furthermore, the absence of a control group precludes any comparison with standard RSI techniques using neuromuscular blocking agents and does not allow conclusions regarding comparative efficacy, safety, or intubating conditions. Although no major complications were observed, the sample size was not sufficient to reliably assess rare adverse events such as pulmonary aspiration. Therefore, the absence of such events should not be interpreted as evidence of safety. However, the study was not designed to compare remifentanil with neuromuscular blocking agents or to establish comparative efficacy, but rather to describe the peri-intubation outcomes associated with a modified RSI approach using remifentanil. Similarly, the outcomes of hypotension and bradycardia were subjective and depended on the decision of treatment by the anesthesiologist, with no predefined threshold. However, as previously indicated, the definition of thresholds is a recurrent debate in pediatrics.^18^

## Conclusion

This study provides additional data describing the peri-intubation outcomes associated with intravenous bolus remifentanil during modified RSI in a selected pediatric population ranging from newborns to adolescents. A first-attempt intubation success rate of 92.9% was observed, and no major complications were reported. However, the observational design, absence of a control group, and limited sample size preclude conclusions regarding comparative safety or efficacy. Larger prospective and controlled studies are required to further evaluate this approach.

## Data availability statement

The individual-level dataset generated and analyzed during the current study originates from our institutional quality assurance database (VAPO) and cannot be made publicly available because of institutional and ethical restrictions. A de-identified dataset, data dictionary, and SAS code used for the analyses may be made available by the corresponding author upon reasonable request, subject to approval by the Institutional Review Board and the data governance policies of Montpellier University Hospital.

## Declaration of generative AI in scientific writing

During the preparation of this work the authors used ChatGPT (OpenAI) in order to improve readability and language. After using this tool, the authors reviewed and edited the content as needed and take full responsibility for the content of the publication.

## Preprint statement

A previous version of this manuscript was posted as a preprint on medRxiv: Pico J, Sola C, Hertz L, Riera J, Scott C, Chapron K, Pirat P, Bringuier S, Dadure C. Remifentanil as an alternative in modified rapid sequence induction: A proof-of-concept study in a selected paediatric population. medRxiv. 2024. 10.1101/2024.12.11.24318829.

## Conflicts of interest

The authors declare no conflicts of interest.
